# Acute physiological effects of the commercially available weight loss/energy product, Fastin-XR^®^, in contrast with the individual effects of caffeine and acacia rigidula

**DOI:** 10.1186/1550-2783-9-S1-P10

**Published:** 2012-11-19

**Authors:** Patrick L Jacobs

**Affiliations:** 1 Superior Performance Research, Miami FL 33186, USA

## Background

The prevalence of overweight and obesity worldwide has resulted in the growth of over the counter weight loss products into one the largest categories of nutritional supplements. However, few commercial products have been properly examined in finished commercial form and seldom have been studied in comparison with individual active ingredients. The purpose of this study was to investigate the acute effects of the commercial weight loss/energy product, Fastin-XR^®^ (High-Tech Pharmaceuticals, Inc., Norcross, GA) on measures of metabolic and hemodynamic activity in comparison with the effects of caffeine and the effects of acacia rigidula.

## Methods

Ten recreationally active men, 28.5 ± 5 years of age, voluntarily participated in this investigation. Study participants completed four 3-hour resting metabolic testing sessions in which four treatment conditions including Fastin-XR^R^ (FAS), 300 mg caffeine anhydrous (CAF), 250 mg acacia rigidula extract (AC), and cellulose placebo condition (PL) were examined in randomized order. Physiological activity was determined in 15 minute intervals immediately prior to and 1hr, 2hrs, and 3 hrs following ingestion. Metabolic activity was determined with open flow spirometry (VO2000, Medgraphics, St. Paul, MN) with outcomes including oxygen consumption (VO_2_), respiratory exchange ratio (RER), minute ventilation (V_E_) and oxygen extraction (VO_2_/V_E_). Hemodynamic activity was examined by measurement of heart rate (HR) and blood pressures (SBP, DBP). Values of metabolic and hemodynamic variables were adjusted into change scores relative to baseline levels. Statistical analyses were conducted using a 4x3 ANOVA for repeated measures with the accepted level of significance set at p<0.05.

## Results

The VO_2_ change scores for 1hr, 2hrs, and hrs post ingestion were significantly greater with FAS (22.1%, 19.3%, 16.5%) compared with P (-2.6%, -1.7%, -2.0%), C (9.9%, 8.5%, 3.5%) and with AC (12.0%, 9.3%, 12.5%). The AC condition produced significantly greater VO_2_ compared with PL at all three time points with CAF displaying values greater than PL at 1hr and 2hrs post ingestion. No significant main or interaction effects were detected in values of RER. The FAS condition produced significantly greater elevations in V_E_ compared with PL at all three time points. Both CAF and AC produced significantly greater V_E_ change scores than PL, at 1hr post ingestion. Values of VO_2_/V_E_ were significantly reduced from baseline at 1hr and 2hrs post with FAS and were significantly lower at 1hr post with CAF while AC produced elevations in VO_2_/V_E_ of 5%, 4%, 7%. The changes in HR were significantly greater with FAS than PL at 2hrs and 3hrs post (9.4 and 11.1bpm) while AC resulted in 2.5 and 4.1 bpm greater HR at 1hr and 2hrs post which were significantly greater than P. FAS produced significantly greater blood pressure changes at all three time points compared with PL (SBP↑33%, 26%, 19%; DBP↑26%, 10%, 15%). Changes in DBP were significantly greater than PL with CAF at 1hr (9.4%) and 2hrs (7.1%). Blood pressures were not significantly affected by AC.

## Conclusions

These findings indicate that resting energy expenditure is significantly enhanced with Fastin-XR^R^, 300 mg caffeine anhydrous, or 250 mg acacia rigidula. Hemodynamic activity (HR, SBP, DBP) is significantly elevated with Fastin-XR^R^ with modest effects displayed with caffeine or acacia.

